# Histopathological spectrum of paediatric diffuse intrinsic pontine glioma: diagnostic and therapeutic implications

**DOI:** 10.1007/s00401-014-1319-6

**Published:** 2014-07-22

**Authors:** Pawel Buczkowicz, Ute Bartels, Eric Bouffet, Oren Becher, Cynthia Hawkins

**Affiliations:** 1 Division of Pathology, The Hospital for Sick Children, 555 University Avenue, Toronto, ON M5G 1X8 Canada; 2 The Arthur and Sonia Labatt Brain Tumour Research Centre, The Hospital for Sick Children, Toronto, Canada; 3 Division of Haematology and Oncology, The Hospital for Sick Children, Toronto, Canada; 4 Division of Pediatric Hematology/Oncology, Duke University Medical Center, Durham, USA; 5 Department of Laboratory Medicine and Pathobiology, Faculty of Medicine, University of Toronto, Toronto, Canada

**Keywords:** DIPG, Astrocytoma, Paediatric, Glioma, H3.3, H3F3A, K27M, ACVR1

## Abstract

Diffuse intrinsic pontine glioma (DIPG) is the main cause of brain tumour-related death in children. In the majority of cases diagnosis is based on clinical and MRI findings, resulting in the scarcity of pre-treatment specimens available to study. Our group has developed an autopsy-based protocol to investigate the histologic and biologic spectrum of DIPG. This has also allowed us to investigate the terminal pattern of disease and gain a better understanding of what challenges we are facing in treating DIPG. Here, we review 72 DIPG cases with well documented clinical history and molecular data and describe the pathological features of this disease in relation to clinical and genetic features. Fifty-three of the samples were autopsy material (7 pre-treatment) and 19 were pre-treatment biopsy/surgical specimens. Upon histological review, 62 patients had high-grade astrocytomas (18 WHO grade III and 44 WHO grade IV patients), 8 had WHO grade II astrocytomas, and 2 had features of primitive neuroectodermal tumour (PNET). K27M-H3 mutations were exclusively found in tumours with WHO grade II–IV astrocytoma histology. K27M-H3.1 and *ACVR1* mutations as well as ALT phenotype were only found in WHO grade III–IV astrocytomas, while *PIK3CA* mutations and *PDGFRA* gains/amplifications were found in WHO grade II–IV astrocytomas. Approximately 1/3 of DIPG patients had leptomeningeal spread of their tumour. Further, diffuse invasion of the brainstem, spinal cord and thalamus was common with some cases showing spread as distant as the frontal lobes. These findings suggest that focal radiation may be inadequate for some of these patients. Importantly, we show that clinically classic DIPGs represent a diverse histologic spectrum, including multiple cases which would fit WHO criteria of grade II astrocytoma which nevertheless behave clinically as high-grade astrocytomas and harbour the histone K27M-H3.3 mutation. This suggests that the current WHO astrocytoma grading scheme may not appropriately predict outcome for paediatric brainstem gliomas.

**Electronic supplementary material:**

The online version of this article (doi:10.1007/s00401-014-1319-6) contains supplementary material, which is available to authorized users.

## Introduction

Paediatric brainstem tumours diffusely infiltrative of the pons—diffuse intrinsic pontine gliomas (DIPG) are the most common type of brainstem glioma, accounting for 60–80 % of these tumours [19]. They are currently the number one cause of brain tumour-related death in children with a median survival of approximately 10 months and <10 % 2-year survival [4, 13]. Due to the location of these lesions, surgical intervention is not an option and biopsies have rarely been conducted, with diagnosis based on radiologic features and clinical findings [21]. However, recent biopsy-based studies of DIPG have been safely conducted and may open the door for future biopsy-informed clinical trials [18]. Radiotherapy is the prevailing mode of treatment; however, it is mainly palliative, and clinical trials over the last few decades have not demonstrated a survival benefit of adjuvant chemotherapy [6]. According to a recent review by Jansen et al. [9], there have been 55 clinical trials since 1984, including 26 between 2007 and 2012, with no improved survival or prognosis when compared to radiation therapy alone.

Several studies into the underlying biology of DIPG over the last few years have demonstrated genetic signatures commonly present in subsets of patients [5, 10, 16, 17, 24, 26]. *TP53* represents one of the most commonly mutated genes described in DIPG, present in 40–77 % of cases [5, 10]. Our group and others have demonstrated a high-frequency heterozygous mutation of histone H3.3 in DIPG at residue K27 [10, 24]. The mutation, resulting in replacement of a lysine with methionine (K27M), is present in approximately 70 % of DIPGs versus 21 % of, largely midline thalamic, supratentorial GBMs [10, 22]. K27 is an important residue that can be either acetylated, methylated or ribosylated on all histone H3 variants [2, 14, 15, 20]. Although the biological consequence of this mutation is still under investigation, given the high frequency of this mutation involving a functionally important, conserved residue, it is likely relevant for tumourigenesis. The roles of K27M-H3.3 in dysregulation of PRC2 complex member EZH2 and global decrease in H3K27me3 have been documented [1, 11]. Moreover, the mutation confers a worse overall survival compared to H3.3 wild-type patients and there are specific copy number alterations associated with the mutation including *PDGFRA* and *PVT1/MYC* locus gains and amplification, which are not seen in the wild-type patients [10]. While our understanding of some of the genetic underpinnings of DIPG has improved over the last few years, the assumption has been that these represent brainstem GBMs and that low-grade histology seen in biopsy specimens represents sampling bias. Here, we describe the histopathological features of 72 DIPGs, the largest DIPG histologic series reviewed to date, and correlate it with clinical data as well as recurrent copy number alteration and mutation status of genes frequently affected in DIPG. These data will be crucial for informing future biopsy-based clinical trials.

## Patients and methods

### Patient cohort

Seventy-two infants and children diagnosed with DIPG between 1984 and 2012 were included in the study. Forty-one were diagnosed and treated at the Hospital for Sick Children and 31 cases were referred from outside. The cooperating pathologists and institutions are listed in acknowledgements. Clinical presentation was consistent with DIPG including history of one or more of progressive headache, vomiting, diplopia, unsteady gait, slurred speech, papilledema, cranial nerve palsies and signs of ataxia. Imaging characteristics were also in keeping with a diffuse pontine tumour, including a contrasting lesion on MRI with diffuse involvement of at least 50 % of the pons required for DIPG diagnosis. Thirty-five of the patients were female and 37 were male. The mean age of diagnosis for our cohort was 6.87 years (median 6.47 years; range 0 days to 15.35 years). More than 80 % of patients were under the age of 10 at the time of diagnosis. The median survival was 0.83 years.

Chemotherapy, if any, varied based on protocols available at the time of presentation or progression and included temozolomide (Temodol), nimotuzumab (TheraCIM), tipifarnib (Zarnestra), retinoic acid, bevacizumab (Avastin), temsirolimus (Torisel) or cituxumab (IMC-A12).

Radiotherapy data were available for 59 patients. Of these, all but 6 patients received focal radiation. Forty-eight patients received a dose of 54 Gy over 30 fractions. One patient received 25 Gy over 5 days. Three patients received 56 Gy and one patient received 59 Gy of radiation. This same patient was re-irradiated. All clinical and molecular features of our DIPG cohort can be found in Table S1.

### Histology and immunohistochemistry

All available surgical and autopsy materials were examined by routine haematoxylin and eosin (H&E) staining and as indicated, by immunohistochemical staining (IHC) for selected proteins (including MIB1, GFAP, p53, MAP2, NeuN, and nestin) using standard automated immunoperoxidase detection. 5 µm sections were cut from paraffin blocks and mounted on positively charged slides. Immunodetection was performed with the automated Benchmark XT stainer (Ventana, Tucson, AZ) using the Ultraview Universal DAB Detection kit (Ventana). Slides were counterstained with haematoxylin II kit (Ventana). All cases were assigned a diagnosis and grade according to WHO criteria. For 44 DIPG patients the entire brain or partial brain specimen with detailed autopsy report was available for examination, allowing extensive histological review of tumour spread and statistical testing related to tumour dissemination. For the cases where entire brain was available, 15–20 blocks were taken including approximately ten sections from the brainstem (medulla, pons and midbrain) as well as sections from the thalamus, basal ganglia, occipital lobe, temporal lobe, frontal lobe and deep white matter. Sections were examined and evaluated for extent of disease and presence of leptomeningeal and/or intraventricular dissemination. An additional nine autopsy cases referred from collaborating pathologists and institutions had insufficient autopsy material to evaluate leptomeningeal dissemination or distant tumour spread, but were evaluable for WHO diagnosis and grade and had material available for molecular diagnosis. A further 19 cases had available surgical pre-treatment material and were only assessed for tumour grade and molecular analysis when sufficient biological material was available. Where applicable, pathological reports from referring pathologists were reviewed to corroborate histopathological diagnosis of reviewed slides.

### Molecular analysis

Fifty-one DIPG patients had sufficient biological material to perform targeted sequencing of *H3F3A*, *HIST1H3B/C*, *PIK3CA,* *TP53* and *ACVR1* using Fluidigm array and ion torrent chips (Life Technologies). An additional 15 samples were sequenced for coding regions of *H3F3A* and *HIST1H3B/C* using Sanger fluorescence sequencing. The histone H3 and *TP53* mutational status of a subset of these patients has previously been reported [10]. *ACVR1* mutations in a subset of these patients were previously described [3]. Alternative lengthening of telomeres was assessed as previously described by either terminal restriction fragment assay (TRF) [8] or C-circles [7].

### Statistical analysis

Where applicable, statistical analysis was performed using SPSS v21 (IBM Corporation) or GraphPad Prism 5 software. Overall survival was estimated using the Kaplan–Meier method and univariate assessments of Kaplan–Meier plots were tested using log rank. Unpaired two-tailed Student’s *t* test was used for statistical testing of continuous scale data. *p* values ≤0.05 were considered to be significant. Multivariate Cox proportional hazard models and significance testing based on the Wald test (*α* = 0.05) were used for multivariate analysis. Statistical testing of leptomeningeal dissemination, histone H3 mutations and histology were employed only for samples with sufficient clinical and molecular data. Statistics on leptomeningeal dissemination was not reported for biopsy patients as tissue specimens were insufficient to determine the presence or absence of leptomeningeal disease.

## Results

### DIPG represents a histologic spectrum with grade not predictive of survival

**Fig. 1:**
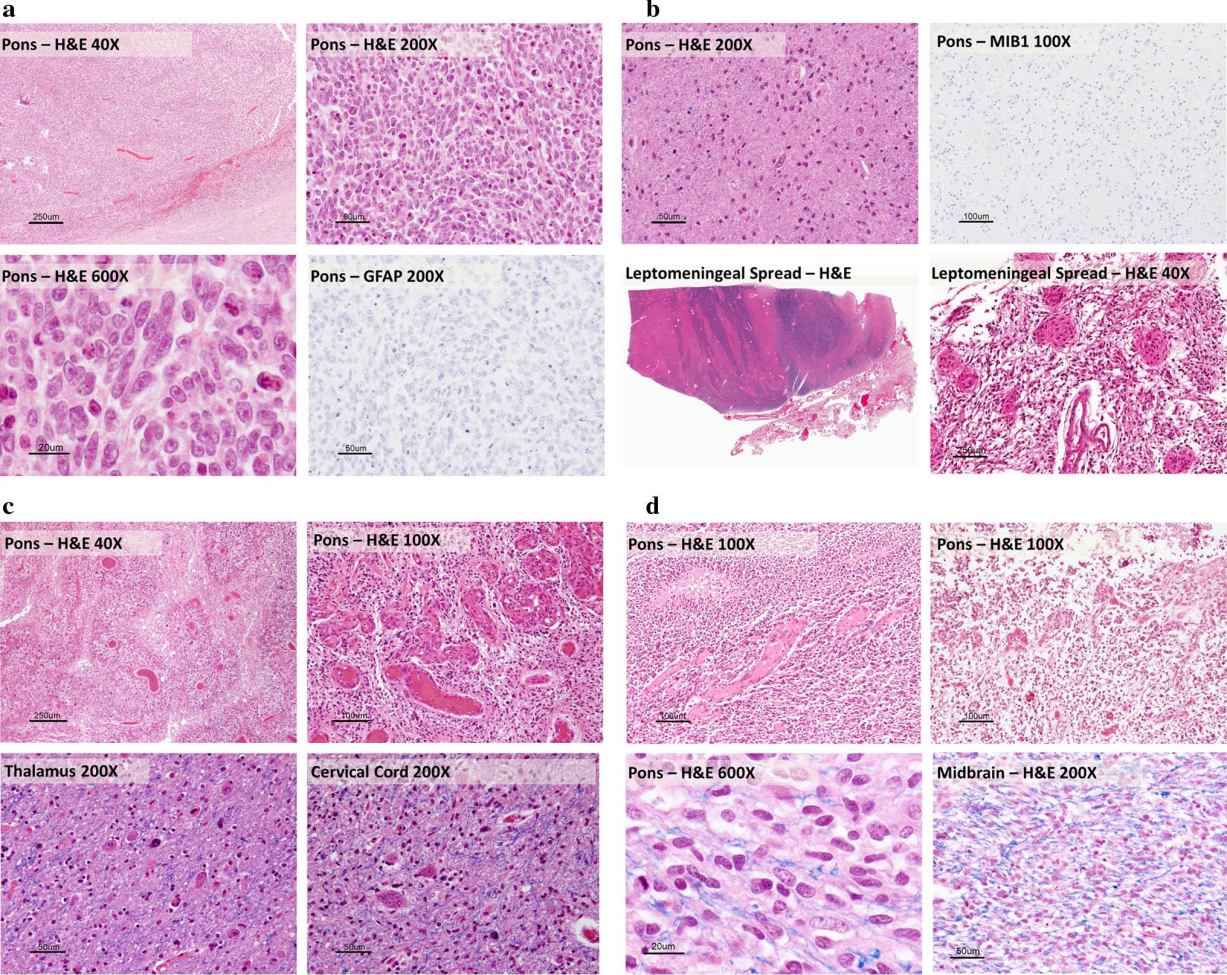
Histologic spectrum of DIPG. **a** PNET, WT-H3: the tumour was not directly invasive and sharply demarcated from adjacent brain (*upper right panel*). On H&E the tumour cells have little cytoplasm with anaplastic nuclei and prominent nucleoli. There was no vascular endothelial proliferation and no pseudopalisading necrosis typical of high-grade astrocytomas. On IHC the tumour cells were negative for GFAP (*lower right panel*). **b** LGA, K27M-H3.3: On H&E this was an infiltrative astrocytic tumour with no high-grade features (WHO grade II). There was no vascular endothelial proliferation, necrosis and very few mitoses (*upper left panel*). Less than 1 % of tumour cells were positive for MIB1/Ki-67 (*upper right panel*). There was leptomeningeal spread of the tumour and an exophytic component involving both the pons and the medulla (*lower panels*). **c** GBM, K27M-H3.3: at autopsy this tumour has classic GBM features throughout the pons (*upper left and right panels*). There was diffuse intraparenchymal tumour infiltration into the thalamus (*lower left panel*) and cervical cord (*lower right panel*). **d** GBM, WT-H3.3: on autopsy, H&E staining revealed histology consistent with that of GBM; including pseudopalisading necrosis (*upper left panel*) and vascular endothelial proliferation (*upper right panel*). There was diffuse intraparenchymal infiltration of the tumour into the midbrain (*lower right panel*)

Upon pathological review, 8 (11 %) samples were found to be diffuse astrocytoma WHO grade II (LGA), 18 (25 %) were anaplastic astrocytoma (AA, WHO grade III), 44 (61 %) were glioblastoma (GBM, WHO grade IV), and 2 (3 %) were primitive neuroectodermal tumours (PNET, WHO grade IV). The PNETs were more sharply demarcated from adjacent brain than the glial tumours, had no vascular endothelial proliferation or pseudopalisading necrosis and were immunonegative for GFAP (Fig. 1a). For 17 of the GBM cases we reviewed the histology at multiple levels of the brainstem as well as the thalamus, frontal cortex and metastatic disease and recorded the histologic grade of the tumour at that level (Table 1). Interestingly, this analysis showed that in 7 (40 %) cases the GBM histology (vascular endothelial proliferation and necrosis) was limited to the pons while other levels showed WHO grade II or III histology. In no case was GBM histology present at all levels of the brain.

**Table 1:** WHO diagnosis of diffuse intrinsic pontine glioma by tumour location

Autopsy cases	Pons	Medulla	Midbrain	Thalamus	Frontal lobe	Mets
DIPG03	GBM	DA	AA	DA	–	–
DIPG04	GBM	AA	AA	DA	–	–
DIPG05	GBM	GBM	AA	AA	–	GBM
DIPG06	GBM	DA	GBM	–	–	GBM
DIPG07	GBM	DA	GBM	AA	–	–
DIPG08	GBM	AA	GBM	–	–	–
DIPG18	GBM	AA	AA	AA	–	–
DIPG19	AA	AA	AA	AA	–	GBM
DIPG24	GBM	AA	AA	–	–	–
DIPG27	GBM	AA	GBM	AA	–	–
DIPG58	GBM	AA	GBM	GBM	DA	–
DIPG59	GBM	GBM	GBM	GBM	AA	–
DIPG60	GBM	DA	AA	–	–	–
DIPG61	GBM	AA	AA	AA	–	–
DIPG62	GBM	AA	AA	DA	–	–
DIPG66	GBM	AA	GBM	GBM	AA	–
DIPG78	GBM	AA	GBM	–	–	–

*GBM* glioblastoma grade IV, *AA* anaplastic astrocytoma grade III, *DA* diffuse astrocytoma grade II

We had only one DIPG patient for whom biopsy and post-mortem material was available. This showed AA histology on biopsy and GBM histology on autopsy. However, as described above it is quite possible to target lower-grade areas on biopsy, thus it is unclear if this represents true progression of disease. Future studies with larger series of matched biopsy/autopsy specimens would need to be conducted in order to determine if anaplastic progression occurs in DIPG between diagnosis and autopsy.

**Fig. 2:**
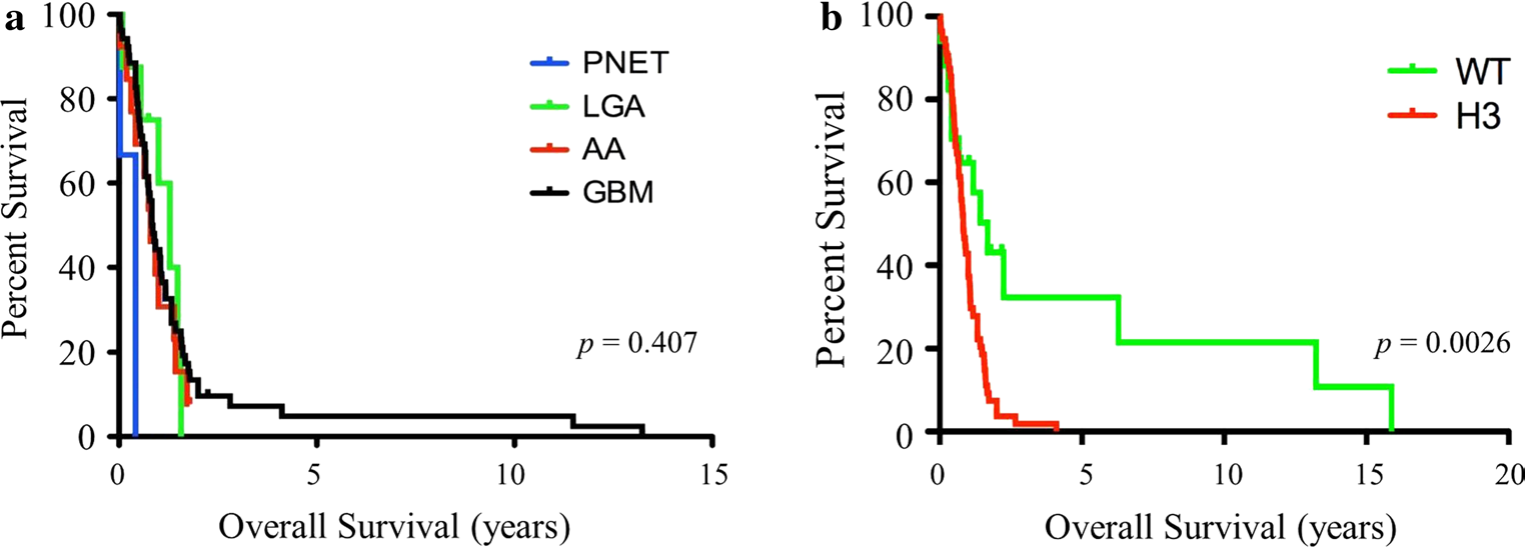
Histone mutations but not histologic grade predicts survival of DIPG patients. **a** Kaplan–Meier analysis of DIPG based on tumour grade and histology reveals there is no significant difference in survival based on histologic diagnosis (*p* = 0.407). **b** DIPG patients with K27M-H3 mutation (*red*) have worse overall survival as compared to wild-type H3 DIPG patients (*green*) as determined by Kaplan–Meier analysis (log rank, *p* = 0.0026)

There was no significant difference in survival based on histology (Fig. 2a, *p* = 0.407). Median survival for patients with diffuse astrocytoma grade II, anaplastic astrocytoma and GBM was 0.88, 0.83 and 0.85 years, respectively. However, the association of age of diagnosis and tumour grade was significant (*p* = 0.040). The age of diagnosis for the glial tumours increased with tumour grade, as LGA, AA, and GBM had mean age of diagnosis at ages 4.19, 6.67 and 7.61 years, respectively. This same trend was observed when biopsy samples and autopsy samples were analysed individually (Table S2).

### DIPGs exhibit leptomeningeal spread and distant spread

For 44 patients sufficient tissue was available to assess extent of spread and the presence of disseminated disease. Seventeen of 44 patients (38.6 %) had leptomeningeal spread at autopsy (for example, see Fig. 1b). Further, 11/44 (25 %) patients had tumour cells present beyond the brainstem (spinal cord and/or thalamic involvement; for example, see Fig. 1c), including four patients with tumour cells diffusely infiltrating as far rostrally as the frontal lobe. There was no statistical correlation between presence of leptomeningeal dissemination and histologic grade. While it did not reach statistical significance, our data suggest that overall survival for patients with leptomeningeal spread was worse, averaging 7.9 months, whereas patients without leptomeningeal spread had an average overall survival of 12.2 months (*p* = 0.09). Of the four cases which had spread to the frontal lobe at autopsy, three were GBM (two K27M-H3 and one WT-H3) and one was AA (WT-H3.3). Interestingly, the mean survival for the K27M-H3 mutant cases was 0.63 years and for the wild-type cases was 1.84 years, although the numbers are too small to make definitive conclusions based on this. Similarly, the small number of patients with this distant spread at autopsy did not allow for statistical analysis of tumour grade and H3-mutational status. Histone mutation status was available for 15 patients with leptomeningeal dissemination, of which 8 (53 %) had the K27M-H3.3 mutation. Gains of *PDGFRA* were detected in 4/8 DIPGs with K27M-H3.3 mutation and leptomeningeal dissemination. *TP53* mutation status was available for 11 patients with leptomeningeal disease. Six of these patients had *TP53* mutations. One DIPG patient with leptomeningeal disease (GBM histology) had a p.Gly328Val substitution in ACVR1 and one patient (LGA histology) had a p.Glu545Gly alteration in PIK3CA. There was no statistical difference in the frequency of *PIK3CA* mutations between disseminated and non-disseminated cases.

### K27M-H3.3 mutations and histology

Tumour tissue from the pons of 66 DIPG patients was screened for K27M mutation in histone H3 as previously described [10]. 42/66 (64 %) were found to be mutated for K27M-H3.3, with an additional eight patients with K27M-H3.1 mutations (12 %). Mutant allele frequencies from deep sequencing suggest histone mutations are heterozygously present in all tumour cells. Of the 14 biopsy tested for histone mutations, 7 (50 %) were mutated for K27M-H3.3 and none had histone H3.1 mutations. Of the 52 autopsy cases tested for histone H3 mutations, 35 (67 %) were mutated for K27M-H3.3 and 8 (15 %) were mutated for K27M-H3.1. We previously performed survival analysis in a subset of these DIPG patients based on histone mutation status [10]. In this larger series, the observation that patients with K27M mutation in either histone H3.1 or H3.3 have worse overall survival compared to patients with no histone mutations remains significant (Fig. 2b, Log rank, *p* = 0.0026). On multivariate analysis (Cox regression), which included histone mutational status, age of diagnosis, histological grade and sex, only histone mutation status was determined to be a significant predictor of overall survival with a hazard ratio of 2.8 (95 % confidence intervals, 1.35–5.78, *p* = 0.006, Table 2). DIPG patients who had wild-type H3 were diagnosed at a significantly younger age than patients with K27M-H3 (wild-type 4.84 years ± 4.13 vs. K27M-H3 7.36 years ± 3.31, *p* = 0.018). There was also correlation between histologic grade and mutational status. Eighty-eight percent of GBMs harboured a K27M mutation in H3 versus 60 % of AAs and 71 % of LGA. K27M-H3.3 was found at a statistically higher ratio in GBM patients (78 %) than patients with AA histology (33 %) (*p* = 0.0016). The association of anaplastic astrocytoma histology and K27M-H3.1 mutations approached significance (*p* = 0.058). All K27M mutant tumours were glial (tumours with PNET histology were wild-type), but represented a spectrum of WHO grades (II–IV). There was no correlation between K27M-mutation status and leptomeningeal spread. Some patients were wild-type for H3.3 yet exhibited features of high-grade astrocytic tumours such as pseudopalisading necrosis, vascular endothelial proliferation and mitoses (Fig. 1d). Interestingly, despite typical high-grade glioma features, patients who were wild-type for H3 with GBM histology survived significantly longer than patients mutated for K27M with GBM histology (mean survival of 1.99 years vs. 0.96 years; *p* = 0.026). Conversely, some DIPG patients exhibited low-grade tumours with low proliferative index, yet were mutated for K27M-H3.3 (Fig. 1b) and exhibited a clinical course typical of high-grade gliomas. DIPG with grade II and III histology carrying the mutation do just as poorly as GBM. The overall survival of K27M-H3.3 tumours with grade II and III histology was 0.82 ± 0.47 years compared to 0.91 ± 0.77 years for K27M-H3.3 grade IV tumours (*p* > 0.05). Patients with WHO grade II diffuse astrocytomas, but wild-type for H3 survived longer than K27M-H3.3 mutant WHO grade II astrocytomas. Histologically, these cases all had similar features of diffusely infiltrating astrocytic tumour with rare to no mitotic figures, low MIB1, diffuse GFAP immunopositivity and rare to no P53 immunopositivity. Most DIPGs were positive for GFAP, with more diffuse staining present in the grade II lesions and no GFAP immunopositivity present in DIPG with PNET histology.

**Table 2:** Multivariate Cox regression analysis

Variables	HR	*p* value	Lower 95 % CI	Upper 95 % CI
K27M-H3	2.793	0.006	1.350	5.777
Histology	0.808	0.222	0.574	1.138
Age Dx	1.016	0.693	0.940	1.097
Sex	1.267	0.412	0.720	2.231

*Age Dx* age of diagnosis, *HR* hazard ratio, *CI* confidence interval

### DIPG histologic subgroups have unique genetic signatures

**Fig. 3:**
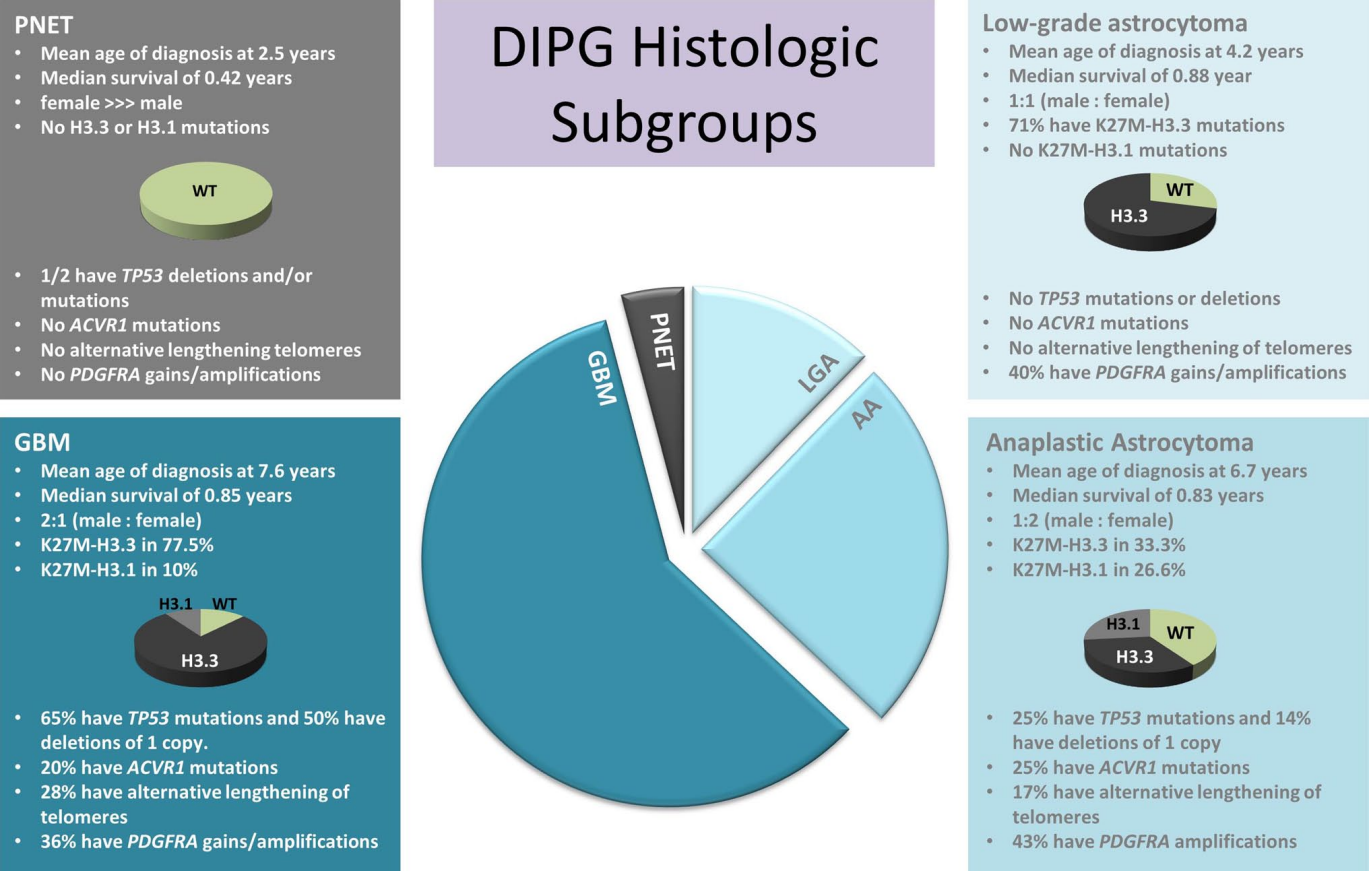
DIPG histologic subgroups have some unique clinical and molecular features including mean age of diagnosis, sex, H3.3 and H3.1 mutations, *TP53* mutations and deletions, *ACVR1* mutations, *PDGFRA* gains/amplifications and alternative lengthening of telomeres (ALT)

DIPG patients, although not significantly differing in their overall survival when based on histology and tumour grade, do show clinical and genetic alterations associated with histology. Sex differences were noted between the different histologies with GBM patients having a 2:1 male to female ratio. This trend was opposite in anaplastic astrocytomas. Mutations and copy number alterations in genes known to be associated with DIPG were also different among histologic subgroups. DIPG with GBM and AA histology had 65 and 25 % incidence of *TP53* mutations, respectively, while no *TP53* mutations were found in low-grade astrocytoma DIPG patient tumours. Immunostaining for P53 did not correlate with *TP53* mutation status, possibly a result of unreliable results from post-mortem tissue. In addition, 50 % of DIPG with both GBM and AA histology had deletion of one copy of *TP53*. As with mutations, no grade II DIPG had this alteration. A similar association was seen with activating mutations of *ACVR1*, where 20 and 25 % of patients with GBM and AA histology had these mutations, respectively, whereas grade II DIPG and DIPG with PNET histology did not harbour *ACVR1* mutations. *PIK3CA* mutations were present in 14.3 % of DIPGs, including WHO grade II-IV astrocytomas but not PNETs. Similarly, *PDGFRA* copy number gains were seen in between 36 and 43 % of patients among all astrocytic histologies of DIPG and not in any PNET. Alternate lengthening of telomeres (ALT) phenotype was seen in 28 and 17 % of GBM and AA patients but not in LGA or PNET DIPG patients (Fig. 3).

## Discussion

Our results highlight the clinicopathological heterogeneity of DIPG and describe the relationship between histology, leptomeningeal dissemination clinical outcome and molecular features. Based on WHO guidelines, DIPG have histology corresponding to grade II, grade III and grade IV tumours. Several cases with typical MRI and symptoms at presentation had histologies on autopsy that would not be considered “classic” for DIPG such as PNET or LGA. Previous case studies have reported DIPG with PNET histology [23]. As in our cases, they described these tumours as having poorly differentiated cells, with scant cytoplasm and round nuclei. Interestingly, on gene expression profiling, these tumours resembled supratentorial PNET [23]. Patients in our series who were found at autopsy to have PNET were diagnosed as DIPG based on standard criteria including MRI and clinical presentation. These patients had clinical course of what is expected in the spectrum for “classical” DIPG and while an argument can be made that PNET tumours of the brainstem should not be included as pontine gliomas, the purpose of our study was to investigate the molecular and histologic spectrum of what is clinically called DIPG. If biopsy becomes standard and diagnosis incorporates histologic and molecular criteria, these entities may be given an alternate designation.

Although there is a wide distribution of tumour grade and histology among DIPG, these alone are not a predictor of survival. A previous clinicopathological study by Yoshimura et al. [25] of 40 diffuse brainstem tumours (33 paediatric and 7 adult) reported a significant difference in overall survival between GBM and AA patients. This difference may be the result of inclusion of adult patients in this cohort. When considering only the paediatric population of the previous study, only four patients with AA and one patient with LGA histology were described and the K27M-H3.3 status, which defines clinically relevant subgroups of DIPG [10], was not known. We found some association between histologic grade (GBM grade IV) and H3.3 mutational status, however, K27M-mutated tumours are found in DIPG with grade II, III and IV astrocytoma histology (Fig. 3). Tumours with lower-grade histology carrying the H3.3 mutation had overall survival comparable to that of grade IV GBM tumours. While patients with high-grade tumours wild-type for H3.3 had overall survival comparable to patients with wild-type low-grade astrocytomas. K27M mutation testing may be helpful for biopsy specimen diagnosis, particularly given the potential histologic intratumoural heterogeneity observed in DIPG, as some tumours with classic GBM histology possess grade II or grade III histology areas, which may be targeted on biopsy. A majority of these heterogeneous cases had features of classic GBM in the pons, including vascular endothelial proliferation and necrosis; however, when sampling the midbrain or thalamus, these features were not present and histology was consistent with LGA or AA. While wild-type status does not imply a better survival (for example, the PNET-like tumours were wild-type for H3.3 and had poor outcome), the presence of a K27-H3 mutation is associated with poor outcome, despite histologic appearance. With advent of biopsy-driven clinical trials [12], K27M mutational status may be pivotal in trial design, particularly when a tumour is classified as a grade II due to limited biopsy specimen.

Distribution of *PDGFRA* gains/amplification as well as *PIK3CA* mutations was similar among all astrocytic tumours, irrespective of grade, whereas ALT phenotype and mutations in *TP53* and *ACVR1* were only observed in tumours with AA and GBM histologies. None of the *PDGFRA* gains/amplifications, *PIK3CA* mutations, ALT phenotype or *ACVR1* mutations were found in tumours with PNET histology.

Thirty-eight percent of patients exhibited leptomeningeal spread of their tumour. The prevalence of leptomeningeal dissemination and distant tumour spread in our series of patients was similar to that reported previously [25]. We found no association with leptomeningeal dissemination and histology. The frequency of certain molecular features common amongst DIPG such as histone H3 mutations, *PDGFRA* gains/amplifications, *ACVR1* and *PIK3CA* mutations were not enriched in patients with leptomeningeal dissemination. Interestingly, none of the patients with leptomeningeal dissemination were found to have ALT phenotype. Diffuse tumour invasion of the brainstem was common among all of the DIPGs; with 25 % diffusely involving the upper cervical cord and thalamus. Some patients exhibited distal spread as far as the frontal lobes. This observation may be critical for clinical decision-making, as focal radiation may be inadequate for patients with leptomeningeal and/or extra-brainstem spread. These patients may benefit from new treatment strategies targeted towards inhibiting tumour infiltration rather than proliferation, especially since some of these very infiltrative tumours exhibit few mitoses and scarce MIB1 positivity. Conversely, on autopsy two patients had severe radiation necrosis of the brainstem with several other patients showing mild to moderate radiation necrosis.

Based on the histologic spectrum of DIPG as well as the prognostic and therapeutic implications of K27M-H3, our findings argue for testing H3-mutation status of biopsy specimens at diagnosis. This important genetic information could be rapidly integrated into the clinical practice, particularly in atypical DIPG, such as those with PNET histology or low-grade lesions with mutant histone H3 that have aggressive clinical course not typically encountered in supratentorial LGA. Importantly, the WHO grading scheme does not predict outcome in DIPG patients. New therapeutic approaches need to incorporate molecular and histological data in order to achieve maximum benefit for DIPG patients.

## Electronic supplementary material

Below is the link to the electronic supplementary material.

Supplementary material 1 (XLSX 11 kb)

Supplementary material 2 (XLSX 10 kb)
